# An intelligent recognition method of chromosome rearrangement patterns based on information entropy

**DOI:** 10.1038/s41598-022-22046-x

**Published:** 2022-11-16

**Authors:** Fushun Wang, Ruolan Zhang, Xiaohua Sun, Junhao Wang, Hongquan Liu, Kang Zhang, Chunyang Wang

**Affiliations:** 1 College of Information Science and Technology, Hebei Agricultural University, Baoding, 071000 People’s Republic of China; 2 Hebei Key Laboratory of Agricultural Big Data, Baoding, 071000 People’s Republic of China; 3 Department of Digital Media, Hebei Software Institute, Baoding, 071000 People’s Republic of China; 4 Department of Urban and Rural Construction, Hebei Agricultural University, Baoding, 071000 People’s Republic of China; 5 College of Life Science, Hebei Agricultural University, Baoding, 071000 People’s Republic of China; 6 State Key Laboratory of North China Crop Improvement and Regulation, Hebei Agricultural University, Baoding, 071000 People’s Republic of China; 7 Hebei Key Laboratory of Plant Physiology and Molecular Pathology, Hebei Agricultural University, Baoding, 071000 People’s Republic of China

**Keywords:** Biological techniques, Computational biology and bioinformatics, Plant sciences

## Abstract

Chromosome rearrangements play an important role in the speciation of plants and animals, and the recognition of chromosome rearrangement patterns is helpful to elucidate the mechanism of species differentiation at the chromosome level. However, the existing chromosome rearrangement recognition methods have some major limitations, such as low quality, barriers to parental selection, and inability to identify specific rearrangement patterns. Based on the whole genome protein sequences, we constructed the combined figure according to the slope of the collinear fragment, the number of homologous genes, the coordinates in the top left and bottom right of the collinear fragment. The standardized combination figure is compared with the four standard pattern figures, and then combined with the information entropy analysis strategy to automatically classify the chromosome images and identify the chromosome rearrangement pattern. This paper proposes an automatic karyotype analysis method EntroCR (intelligent recognition method of chromosome rearrangement based on information entropy), which integrates rearrangement pattern recognition, result recommendation and related chromosome determination, so as to infer the evolution process of ancestral chromosomes to the existing chromosomes. Validation experiments were conducted using whole-genome data of *Gossypium raimondii* and *Gossypium arboreum*, *Oryza sativa* and *Sorghum bicolor*. The conclusions were consistent with previous results. EntroCR provides a reference for researchers in species evolution and molecular marker assisted breeding as well as new methods for analyzing karyotype evolution in other species.

## Introduction

Chromosome rearrangement is an important driving force for the generation of new genes, adaptive enhancement, functional gene evolution, and the formation of new species^1–5^. Karyotypic changes at a given ploidy level are mediated by chromosome rearrangements such as insertions, duplications, deletions, inversions and translocations altering the size and morphology of chromosomes^6–8^. With the emergence of new technologies, various new methods have been successively applied to chromosome rearrangement research. For example, a previous study used fluorescence in situ hybridization (FISH) to explore the phenomenon of chromosome rearrangement in *Silene latifolia*, which suggest that chromosome rearrangement is an integral part of sex chromosome evolution^9^. Some research discovered chromosome rearrangements in the *Lolium* and *Avena sativa L.* genomes by means of genetic mapping^10^. The potential chromosome rearrangements of *Mimulus lewisii* and *Mimulus cardinalis* were identified by comparing the maps^11^. Many researchers identified chromosome rearrangements in cataract and glaucoma by comparative genomic hybridization (CGH) microarray approach^12^.

Karyotype evolution is an important issue in species evolution research. Some studies reconstruct the ancestral genome by looking for regions of collinearity, but does not explain the reason for the reduction in chromosome number or the evolution of karyotypes from ancestral chromosomes to extant chromosomes^13–17^. Some discusses the biological mechanism of chromosome reduction, but does not explain how small chromosomes are formed^18^. And others discover important role of telomeres in chromosome rearrangement^19^. A telomere-centered chromosome rearrangement theory, which established a unified model of chromosome number reduction and emphasized the important role of telomeres in chromosome rearrangement^20^. The theory explains karyotype evolution in monocots brachypodium^21^, rice^22^, corn and sorghum^20^, camelina^23^, celery^24^, and may also elucidate karyotype evolution in yeast^25^, vertebrate^26^.

It is difficult and inefficient to detect the large-scale rearrangements using fluorescence in situ hybridization. Other methods also have shortcomings. Genetic map making has certain restrictions on parental and progeny population selection. High-throughput sequencing technology requires professional operation and is expensive. Comparing maps can detect chromosome rearrangements efficiently and quickly, but further analysis is required for specific rearrangement patterns.

In response to the above existing problems, this study performed sequence alignment based on the whole genome protein sequences of *Brassica rapa*. Through the whole genome collinearity analysis, on linear chromosomes, the homologous gene dot map between two genomes was built. The main purpose of EntroCR method is to detect four chromosome rearrangement patterns, those are inner-inner joining (CIIJ), inner-end joining (CIEJ), end-end joining (CEEJ), and nested chromosome fusions (NCF)^27–30^. According to a certain combination strategy, EntroCR combines the information entropy analysis strategy to compare the standardized combination map with the pattern library. Finally, we identified specific rearrangement patterns and associated chromosomes, and further inferred evolutionary processes.

## Materials and methods

### Data sources

Whole genome CDS sequences (CDS files), protein sequences (PEP files), and annotation files (GFF files) of five species were downloaded from related database (Table 1).

**Table 1:** Genomic data information.

Latin name	Abbreviation	The number of chromosomes	Data sources
*Brassica rapa*	Bra	10 * 2	http://brassicadb.org/
*Gossypium raimondii*	Gra	13 * 2	https://phytozome.jgi.doe.gov/pz/portal.htm
*Gossypium arboreum*	Gar	13 * 2	https://cottonfgd.org/about/download.html
*Oryza sativa*	Osa	12 * 2	http://chibba.Agtec.Uga.edu/
*Sorghum bicolo*	Sbi	10 * 2	http://jgi.doe.gov/

### Preprocess genomic data

In order to extract target data from the genomic sequences and annotation files, the downloaded genomic data was processed with a custom python script to obtain the blast results, which is convenient for subsequent research and analysis. The information for downstream analysis was extracted from the genome annotation files, which include chromosome number, gene start and end positions, gene transcription direction, and gene ID information. Gene ID was renamed and numbered according to the order of genes on chromosomes. The ID in the CDS and protein sequence file is corresponding to the new ID of the gene in the annotation file. The processed genomic data is annotated with a uniform nomenclature.

### Search homologous sequence

BLASTp algorithm was used to search for homologous gene pairs between species. Gene pairs with the expected value (E-value) not greater than 10–5 and score evaluation (Score) higher than 100 were retained, so that the subsequent genome collinearity analysis results are more reliable.

### Draw the ***K***_***S***_ dotplot of homologous genes

The *K*_*S*_ dot plot was drawn by the comprehensive analysis software of WGDI (whole-genome duplication integrated analysis)^31^.The WGDI uses MAFFT^32^ or MUSCLE^33^ to perform multiple sequence alignment, and calculates the synonymous substitution rate by the yn00^34^ or ng86^35^ program of the PAML package. Finally, the visualization is realized by extracting block, and then output blockinfo file. In order to simulate the method in this paper, *Brassica rapa* genome blockinfo file was reconstructed to obtain the simulated species Bra-1 and Bra-2.

### Chromosome rearrangement analysis algorithm

**Figure 1:**
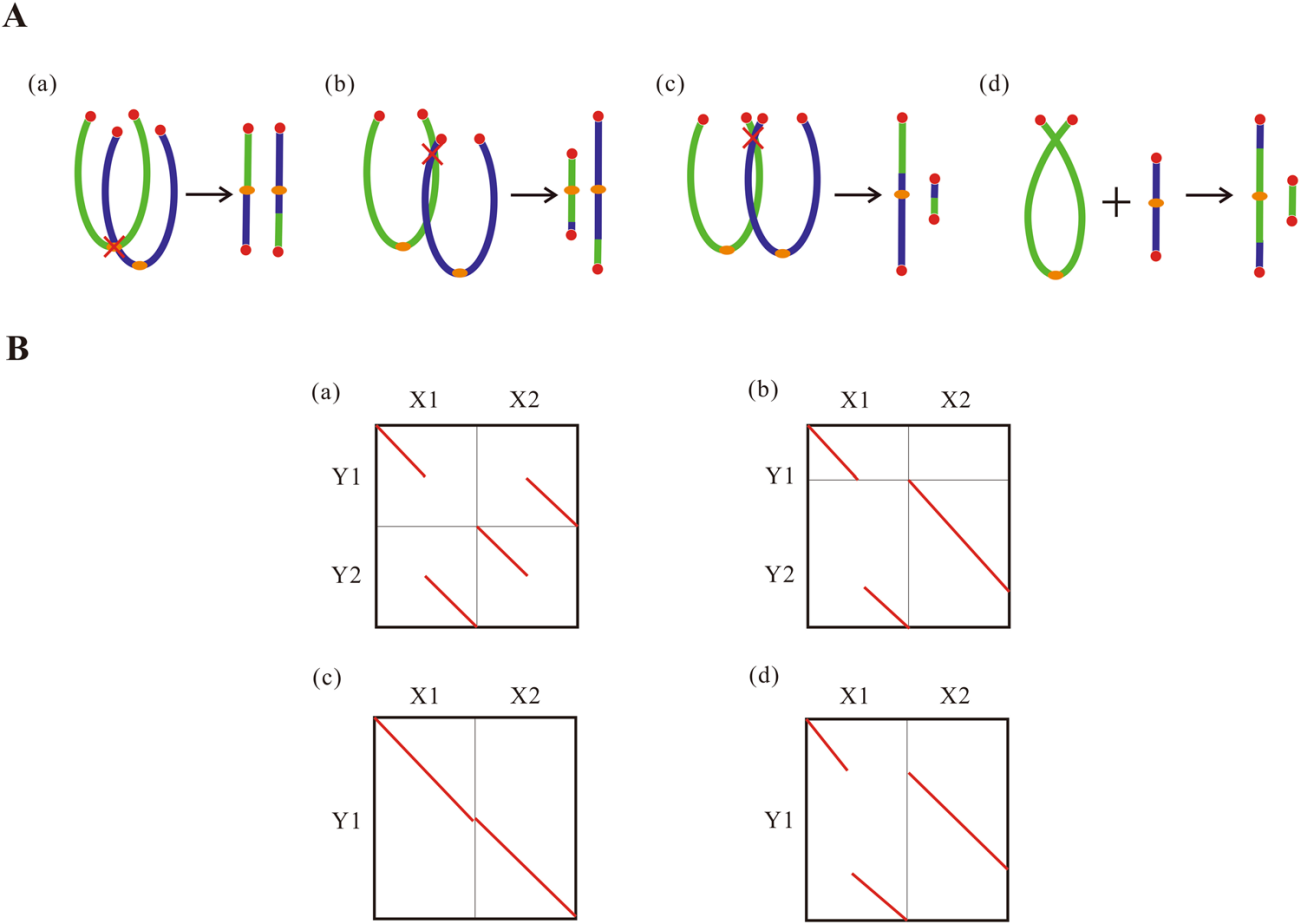
The basic forms of chromosome rearrangement, modified from Meng^27^. (**a**) CIIJ (chromosome inner-inner joining). (**b**) CIEJ (chromosome inner-end joining). (**c**) CEEJ (chromosome end-end joining). (**d**) NCF (nested chromosome fusions).

Information entropy^36^ is one of the objective weighting methods. Its basic idea is to determine the index weight according to the degree of variation of the index. It can fully exploit the potential information of the data and reduce the interference of artificial factors as much as possible, so that the results are more objective and effective. We use the information entropy method to identify four basic forms of chromosome rearrangements (Fig. 1).

The comparison result of two chromosomes from the target species and the reference species is shown as a cell on the *K*_*S*_ dotplot, that is, a comparison unit. In order to combine the comparison units, WGDI was used to get the *K*_*S*_ dotplot of each comparison unit, and read the name of the *K*_*S*_ dotplot. After iteration, we combined a single comparison unit horizontally to obtain a combined graph formed by two comparison units, called image2. Then combinatorial algorithms output a non-blank, binarized combined graph, named binary. On the basis of binary, we performed vertical combination to obtain a combination graph formed by four comparison units, and output a non-blank combination graph, named image4.
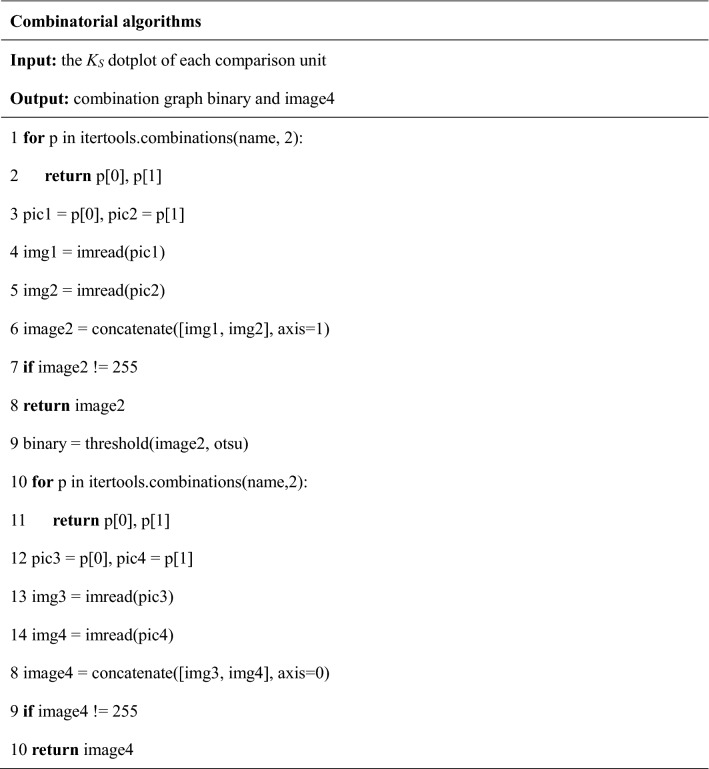


To extract similar features from combination graph, the algorithm readed the width and height of the upper left corner of the combination graph, named top_left_img. The algorithm swapped the left and right of the combination graph formed by two comparison units to obtain swap1; swapped the combination graph formed by four comparison units from left to right to obtain swap2, and swapped up and down to obtain swap3. In order to facilitate the comparison with the characteristics of the standard image: slope, number of homologous genes, and coordinates of the upper left and lower right corners, we changed the sizes of the five forms of binary, swap1, image4, swap2, and swap3. And the algorithm selected the form with the smallest difference from the standard picture parameters and wrote it to the result.csv file.
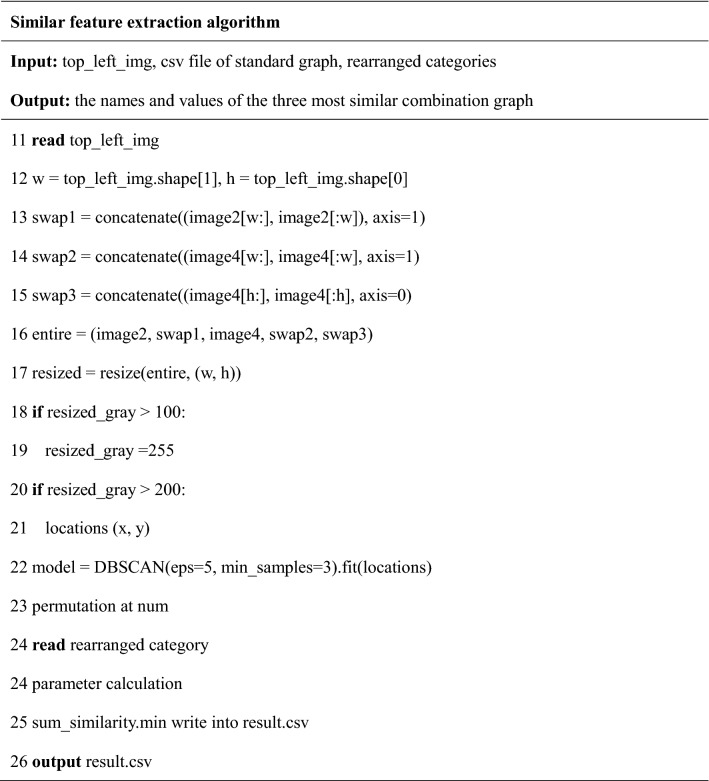


Finally, the algorithm read the result.csv file and used the information entropy method to obtain the similarity of the combination map. We can select the best result among the three combinations with the highest output similarity.

### Information entropy

The python script we wrote implements information entropy calculation as well as outputs similarity.csv, the names and values of the three most similar combination graph. The range of information entropy is [0, 1], and the range of similarity is related to the number of samples.

1. Data normalization. $r_{ij}$ represents the standard value of the ith row recorded under the jth indicator.
  $$ r_{ij} = \frac{{\mathop {\max }\limits_{j} x_{ij} - x_{ij} }}{{\mathop {\max }\limits_{j} x_{ij} - \mathop {\min }\limits_{j} x_{ij} }} $$
2. Calculate the standard value proportion. Get the proportion $p_{ij}$ of the ith row recorded under the jth indicator.
  $$ p_{ij} = \frac{{r_{ij} }}{{\mathop \sum \nolimits_{i = 1}^{n} r_{ij} }} $$
3. Defining information entropy. In a problem of m metrics, n records, the entropy value $h_{j}$ of the jth metric is defined as:
  $$ h_{j} = - \frac{{\mathop \sum \nolimits_{i = 1}^{n} p_{ij} \ln p_{ij} }}{\ln n} $$
  It is stipulated that when $p_{ij} = 0$, $p_{ij} \ln p_{ij} = 0$, then $h_{j} \in \left[ {0, 1} \right]$.
4. Calculate the weight of the jth indicator.
  $$ w_{j} = \frac{{1 - h_{j} }}{{\mathop \sum \nolimits_{j = 1}^{m} \left( {1 - h_{j} } \right)}} $$

## Results

### Rearranged pattern search simulation

**Figure 2:**
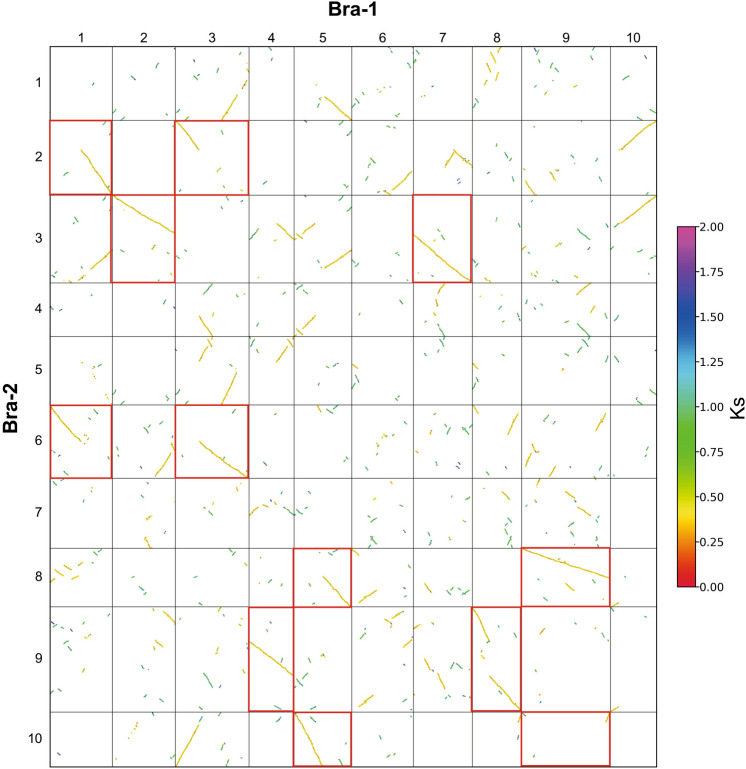
*K*_*S*_ dotplot between Bra-1 and Bra-2. The red boxes represent the chromosomes involved in the four basic rearrangement patterns.

Based on the blockinfo file of *Brassica rapa* genome, in order to construct a *K*_*S*_ dotplot containing four patterns by WGDI (Fig. 2), we synthesized Bra-1 and Bra-2. The target species is Bra-1 and the reference species is Bra-2, forming a total of 10 * 10 comparison units. Based on the comparison units, the search simulation of the rearrangement pattern is carried out. The feasibility of the EntroCR model was evaluated by simulation to determine whether the model could accurately detect the chromosome rearrangement patterns, that is, the four patterns mentioned above.

**Figure 3:**
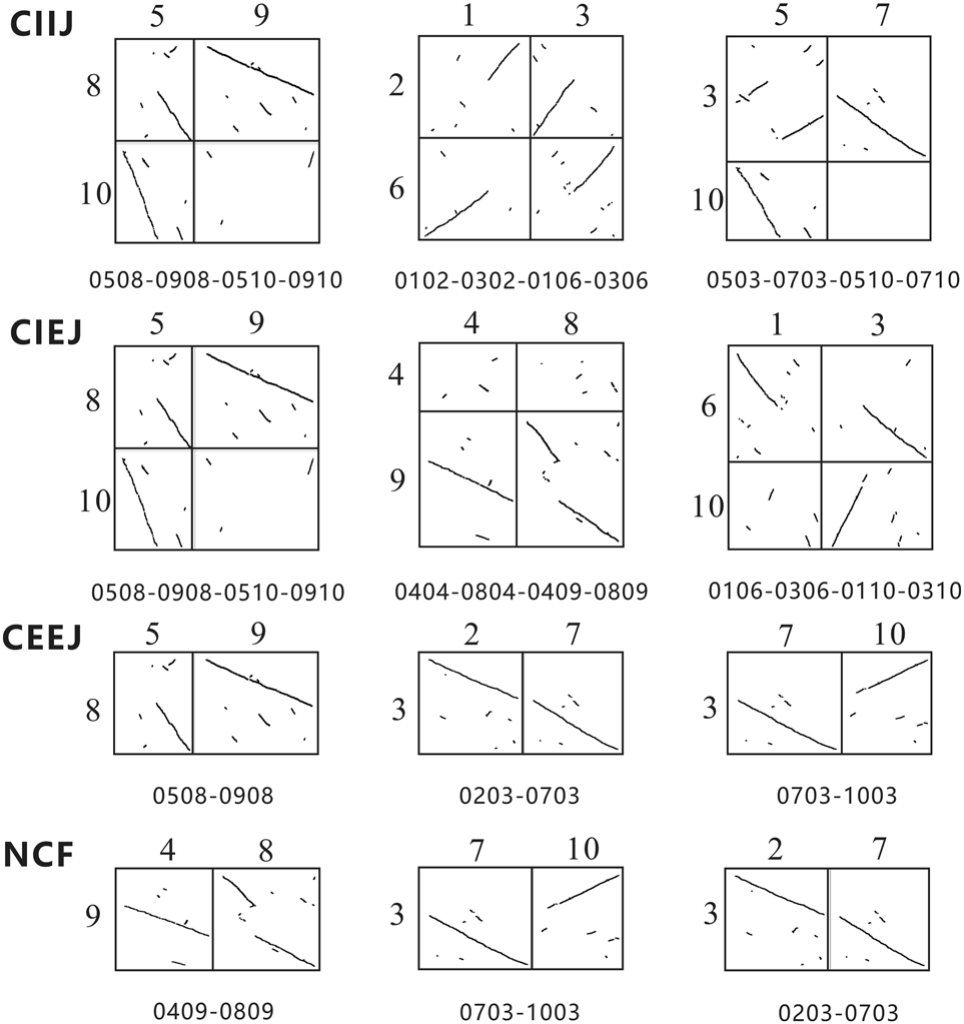
Search results of chromosome rearrangement pattern in *Brassica rapa* reconstruction genome. Note: The combination name is formed according to the clockwise direction of the comparison units, the comparison unit name consists of horizontal and vertical chromosome numbers.

Using the EntroCR model, the number of combinations formed by two comparison units is 450, and the number of combinations formed by four comparison units is 1620. EntroCR model was used to search the rearrangement pattern of four comparison units and two comparison units respectively. And the three combinations with the highest similarity are obtained (Fig. 3), the similarity values are as follows (Table 2).

**Table 2:** Comparison of similarity between different combinations of *Brassica rapa* reconstructed genome.

CIIJ	CIEJ	CEEJ	NCF
2.661	2.195	1.501	2.488
2.578	2.127	1.397	2.322
2.575	2.122	1.357	2.309

In CIIJ pattern, the range of similarity obtained by different combinations is [1.331, 2.661]. The combination of 0102-0302-0106-0306 detected by the model is our constructed CIIJ pattern, and the similarity is 2.578. The crossover between two chromosomes results in the translocation of the chromosome arms to each other. Chromosomes 1 and 3 of Bra-1 crossover, causing the first arm of chromosome 1 to combine with the second arm of chromosome 3 to form Bra-2's chromosome 2, while the other two parts combine to form Bra-2's number chromosome 6. The combination of 0102-0302-0106-0306 is upside-down compared to the standard pattern. The 0508-0908-0510-0910 had the highest similarity of 2.661, but the number of collinear fragments did not match the standard CIIJ pattern; the 0503-0703-0510-0710 combination did not match the number and positions of the collinear segments in the standard CIIJ pattern.

In CIEJ, the range of similarity obtained by different combinations is [1.351, 2.195]. The combination of 0508-0908-0510-0910 detected by the model is our constructed CIEJ pattern, and the similarity is 2.195. This is when one chromosome crosses with another near the telomere to form a short chromosome and a long chromosome. The part of chromosome 5 of Bra-1 species forms chromosome 8 of Bra-2 species, while another part forms chromosome 10 of Bra-2 species with chromosome 9. The combination of 0508-0908-0510-0910 is upside-down compared to the standard pattern. There is a big gap between the similarity of the combination of 0404-0804-0409-0809, 0106-0306-0110-0310 and the combination of 0508-0908-0510-0910.

In CEEJ, the range of similarity obtained by different combinations is [0.512, 1.501]. The combination of 0203-0703 detected by the model is our constructed CEEJ pattern and the similarity is 1.397. That is, chromosome end-end joining. Chromosomes 2 and 7 of Bra-1 join to form chromosome 3 of Bra-2. The 0508-0908 combination has the highest similarity, which is 1.501, but the position of the collinear segment is quite different from the standard pattern; the direction of the collinear segment of the 0703-1003 combination is obviously different from the standard pattern.

In NCF, the range of similarity obtained by different combinations is [1.320, 2.488]. The combination of 0409-0809 detected by the model is our constructed NCF pattern and the similarity is 2.488. That is, nested chromosome fusions. Chromosome 4 of Bra-1 is inserted into chromosome 8 to form Bra-2's chromosome 9. The combination of 0409-0809 is upside-down compared to the standard pattern. The number of collinear fragments for the 0703-1003 combination and the 0203-0503 combination is significantly different from the standard pattern.

From the constructed *Brassica rapa* genome data, EntroCR found the combination of four patterns respectively, among which 0102-0302-0106-0306 is CIIJ pattern, 0508-0908-0510-0910 is CIEJ pattern, 0203-0703 is CEEJ pattern, 0409-0809 is NCF pattern. In addition, other combinations are quite different from the standard pattern, which verifies the validity of the model and shows that EntroCR has a certain search performance.

### Search for rearrangement patterns of *Gossypium raimondii* and *Gossypium arboreum*

**Figure 4:**
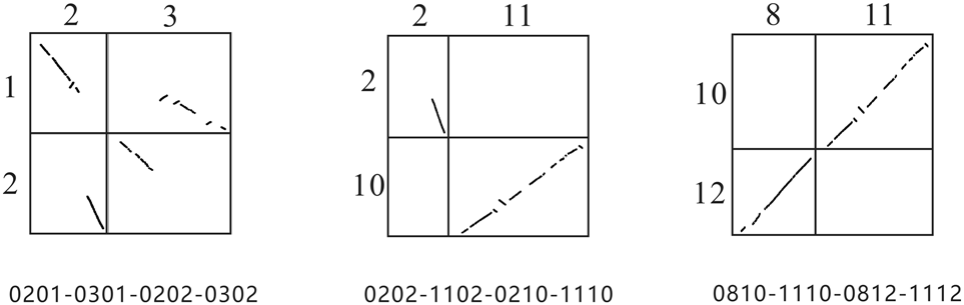
Search results for chromosome rearrangement patterns in the *Gossypium raimondii* and *Gossypium arboreum* genomes.

The *K*_*S*_ dotplot between the genomes of *Gossypium raimondii* and *Gossypium arboreum* is selected as the research object. The numbers on the abscissa axis represent the chromosome number of *Gossypium raimondii*, the numbers on the ordinate axis represent the chromosome number of *Gossypium arboreum*, and the genes on the chromosomes are arranged in order. A total of 13 * 13 comparison units are formed. By using the EntroCR model, the number of combinations formed by two comparison units is 199, and the combination formed by four comparison units is 147. The EntroCR model performed on combinations formed by four comparison units to get the three combinations with the highest similarity (Fig. 4).

The range of similarity obtained by different combinations is [1.426, 3.258]. The model detected the combination of 0201-0301-0202-0302 as the crossover between the chromosomes of *Gossypium raimondii* and *Gossypium arboreum* resulting in the mutual translocation of chromosome arms, that is, the CIIJ pattern, with a similarity of 3.258. The crossover of chromosomes 2 and 3 of *Gossypium raimondii* resulted in the combination of the first half of chromosome 2 and the second half of chromosome 3 to form chromosome 1 of *Gossypium arboreum*, while the other two parts combined to form the chromosome 2 of *Gossypium arboreum*. The 0202-1102-0210-1110 and 0810-1110-0812-1112 combination are significantly different from the standard CIIJ pattern, and their similarity is 3.151 and 3.143, respectively. This result is consistent with previous research^27^.

### Search for rearrangement patterns of *Oryza sativa *and *Sorghum bicolo*

**Figure 5:**
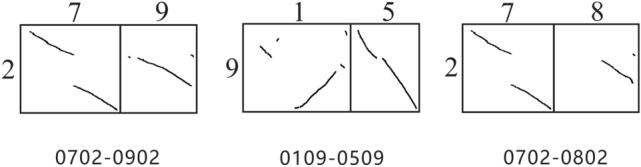
Search results for chromosome rearrangement patterns in the *Oryza sativa* and *Sorghum bicolo* genomes.

The *K*_*S*_ dotplot between the genomes of *Oryza sativa* and *Sorghum bicolo* is selected as the research object. The numbers on the abscissa axis represent the chromosome number of *Oryza sativa*, the numbers on the ordinate axis represent the chromosome number of *Sorghum bicolo*, and the genes on the chromosomes are arranged in order. A total of 12 * 10 comparison units are formed. Using the EntroCR model, the number of combinations formed by two comparison units is 421, and the combination formed by four comparison units is 945. The EntroCR model performed on combinations formed by two comparison units to get the three combinations with the highest similarity (Fig. 5).

The range of similarity obtained by different combinations is [1.153, 2.593]. The model detected the case where the combination of 0702-0902 was a nested chromosome fusion, that is, the NCF pattern, with a similarity of 2.593. The chromosome 7 of *Oryza sativa* is inserted into chromosome 9 to form *Sorghum bicolo* chromosome 2. The 0109-0509 combination is significantly different from the collinear segment position of the standard NCF pattern, with a similarity of 2.567. The 0702-0802 combination has the smallest similarity of 2.451, and the *K*_*S*_ value between the collinear segments has a large difference. This result is consistent with previous research^37^.

## Conclusions

Whole-genome doubling greatly increases spatial and interaction complexity in the nucleus, resulting in the probability of chromosome rearrangements^38^. The previous research methods are not only inefficient but also have limitations on the selection of parental and progeny population. Due to the high cost, the technique requires high expertise, making it difficult to analyze specific rearrangement patterns. In this study, based on the modified whole genome data of *Brassica rapa*, using the information entropy method and referring to the telomere-centered chromosome rearrangement mechanism, the automatic processing of chromosome karyotype analysis was realized. Through the chromosome rearrangement analysis model EntroCR, according to the combined similarity, we performed specific analyses of chromosome rearrangement patterns, identified associated chromosomes, and inferred their evolutionary processes. The method improves the recognition efficiency of rearrangement patterns, reduces the dependence on prior knowledge, and solves the limitations of the human eye in visual space. An effective method provides an important basis for discussing the classification of plants^39,40^, and provide molecular and cytogenetic basis for hybrid improvement and new variety breeding^41^.

## Data Availability

The original contributions presented in the study are included in the article, further inquiries can be directed to the corresponding author/s. The source code and data are available at https://github.com/Emma6674/EntroCR.
